# Sure to Have Support

**Published:** 1897-07

**Authors:** 


### SURE TO HAVE SUPPORT.

THE movement of Secretary of Agriculture Wilson, through our foreign representatives, to remove some of the barriers raised against our live-stock and animal food-products will meet with the warmest approval of the people of our entire land. We are to-day the growers and shippers of the best animal food-products of the world. Our Western prairies are practically free from diseases harmful to meat-products. Our disposition is to send only the best we have for foreign consumption, and our system of sanitary inspection of all animals and their products which are sent abroad has attained such a state of perfection that there can no longer be but one reason why these barriers are maintained, and that is that they do not want our products at all, if they can trump up anything in the nature of an excuse to keep them out. According to their own statistics, their own live-stock at home is far more diseased than ours, and their system of general inspection is no better than our own, and we are not disposed to longer stand and see our products shut out and the exports of these countries landed in profusion on our shores without an objection being raised. Let every veterinarian sustain Secretary Wilson to the utmost, as these embargoes are injuries to our own welfare, not to speak of the unjust reflection on the best veterinary sanitary service our government has been fortunate to have in its employ.

